# Application of random survival forest to establish a nomogram combining clinlabomics-score and clinical data for predicting brain metastasis in primary lung cancer

**DOI:** 10.1007/s12094-024-03688-x

**Published:** 2024-09-03

**Authors:** Zhongxiang Shi, Yixin Chen, Aoyu Liu, Jingya Zeng, Wanlin Xie, Xin Lin, Yangyang Cheng, Huimin Xu, Jialing Zhou, Shan Gao, Chunyuan Feng, Hongxia Zhang, Yihua Sun

**Affiliations:** 1 Department of Clinical Laboratory, Harbin Medical University Cancer Hospital, Harbin, 150081 Heilongjiang China; 2 Imaging Center, Harbin Medical University Cancer Hospital, Harbin, 150081 Heilongjiang China

**Keywords:** Lung cancer, Brain metastasis, Clinlabomics, Clinlabomics-score, Nomogram, Random survival forest

## Abstract

**Purpose:**

To establish a nomogram for predicting brain metastasis (BM) in primary lung cancer at 12, 18, and 24 months after initial diagnosis.

**Methods:**

In this study, we included 428 patients who were diagnosed with primary lung cancer at Harbin Medical University Cancer Hospital between January 2020 and January 2022. The endpoint event was BM. The patients were randomly categorized into two groups in a 7:3 ratio: training (*n* = 299) and validation (*n* = 129) sets. Least absolute shrinkage and selection operator was utilized to analyze the laboratory test results in the training set. Furthermore, clinlabomics-score was determined using regression coefficients. Then, clinlabomics-score was combined with clinical data to construct a nomogram using random survival forest (RSF) and Cox multivariate regression. Then, various methods were used to evaluate the performance of the nomogram.

**Results:**

Five independent predictive factors (pathological type, diameter, lymph node metastasis, non-lymph node metastasis and clinlabomics-score) were used to construct the nomogram. In the validation set, the bootstrap C-index was 0.7672 (95% CI 0.7092–0.8037), 12-month AUC was 0.787 (95% CI 0.708–0.865), 18-month AUC was 0.809 (95% CI 0.735–0.884), and 24-month AUC was 0.858 (95% CI 0.792–0.924). In addition, the calibration curve, decision curve analysis and Kaplan–Meier curves revealed a good performance of the nomogram.

**Conclusions:**

Finally, we constructed and validated a nomogram to predict BM risk in primary lung cancer. Our nomogram can identify patients at high risk of BM and provide a reference for clinical decision-making at different disease time points.

## Introduction

In 2024, lung cancer will emerge as the leading cancer type, with the highest number of new mortalities and the third highest number of new cases [1]. With disease progression, lung cancer eventually metastasizes, with brain metastasis (BM) being the most common metastasis type, and BM risk will gradually increase over time [2, 3]. In addition, lung cancer is the most common primary tumor site for metastatic brain tumors [4]. Approximately 60% of patients with small cell lung cancer (SCLC) will experience BM within 2 years [5], and approximately 10% of patients with non-small cell lung cancer (NSCLC) will experience BM during the initial diagnosis [6]. A study revealed that the median overall survival of patients with BM in NSCLC and SCLC is 7.0 and 4.9 months, respectively. Furthermore, it suggested that BM occurrence can seriously affect disease prognosis and pose a huge burden to patients and their families [7]. Therefore, accurately predicting the probability of BM at different disease time points is extremely vital for patients with primary lung cancer without BM; thereafter, early clinical observation and intervention can be conducted to improve prognosis.

Recently, rapid progress has been observed in omics research, including genomics, proteomics, immunohistochemistry, and radiomics. In 2022, Wen et al. proposed the concept of clinlabomics, a new method that utilizes artificial intelligence (AI) technology to analyze multiple laboratory test results and provide guidance for decision-making in clinical settings [8]. At present, researchers have used AI technology to analyze laboratory test results and construct nomograms [9]. However, in most of the existing studies, laboratory test results have been analyzed along with clinical data; therefore, the problem of collinearity could not be avoided. Therefore, we attempted to independently analyze laboratory test results based on clinlabomics by generating clinlabomics-score to transform complex laboratory test results into an independent predictive indicator. This approach will entirely improve the predictive role of laboratory test results as well as solve the issue of collinearity between them and the clinical data.

At present, AI methods have been applied to establish nomograms for lung cancer [10]. Random survival forest (RSF) is a machine learning method derived from random forest and based on decision trees. It belongs to the AI category and is commonly used in nomograms with time data. RSF can help analyze several variables, prevent overfitting via cross-validation, and select the variables associated with the outcome event [11, 12]. Studies have verified the stable effects of RSF in fields such as gastric cancer and liver cancer [13, 14].

Therefore, in this study, using RSF, we combined clinlabomics-score and clinical data and constructed a nomogram to predict BM risk in primary lung cancer at different times.

## Materials and methods

### Patients

**Fig. 1:**
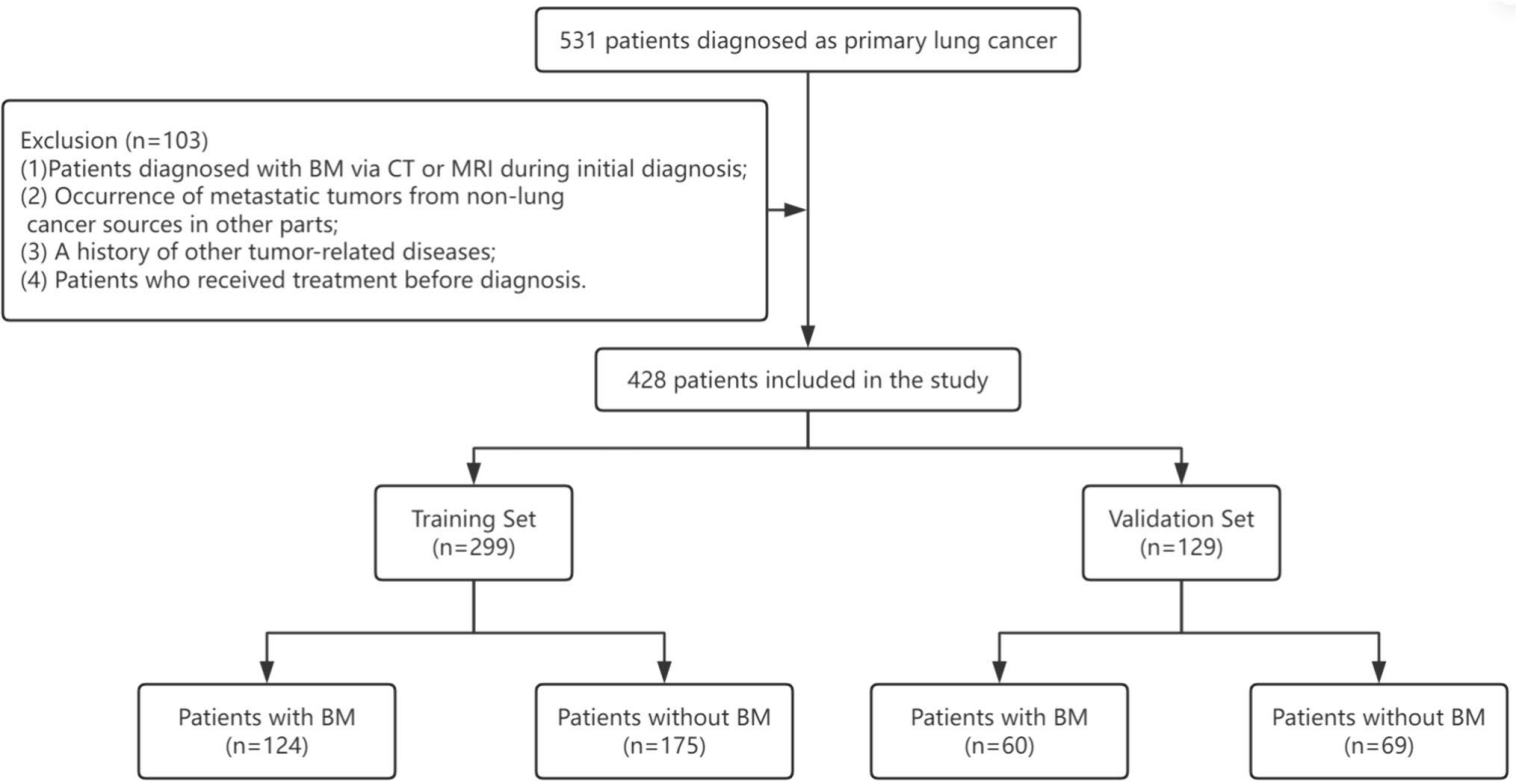
Process of patient selection

The data of patients who were diagnosed with lung cancer at Harbin Medical University Cancer Hospital (Heilongjiang Province, China) between January 2020 and January 2022 were retrospectively analyzed. The patient diagnostic criteria were based on the Guidelines for the Diagnosis and Treatment of Primary Lung Cancer issued by the National Health Commission of the People’s Republic of China in the corresponding year. The inclusion criteria were as follows: (1) patients pathologically diagnosed with lung cancer; (2) lung cancer is a primary tumor; and (3) patients who underwent regular brain computed tomography (CT) or magnetic resonance imaging (MRI) during the disease course. The exclusion criteria were as follows: (1) patients diagnosed with BM via CT or MRI during initial diagnosis; (2) occurrence of metastatic tumors from non-lung cancer sources in other parts; (3) a history of other tumor-related diseases; and (4) patients who received treatment before diagnosis. Finally, 428 eligible patients were included in this study. Figure 1 illustrates the patient selection process.

### Statistical methods and study design

Based on a 7:3 ratio, the included patients (*n* = 428) were randomly divided into two groups: training (*n* = 299) and validation (*n* = 129) sets. First, the clinlabomics-score of the patients in the training set were determined. For this, least absolute shrinkage and selection operator (LASSO) was utilized to analyze the laboratory test results, including blood routine examination, coagulation function examination, biochemical series, and tumor markers. Non-zero coefficient variables were used to establish the clinlabomics-score via coefficient weighting. The clinlabomics-score were calculated for all patients. Subsequently, RSF was used in the training set to analyze the clinlabomics-score and nine other clinical variables. Only relevant variables were included in multivariate Cox regression analysis. Finally, five independent predictive factors were selected for BM. A nomogram was constructed to predict the probability of the patients who will not experience BM (Non-BM Prob) at 12, 18, and 24 months after initial diagnosis. Bootstrap C-index and area under the curve (AUC) were used to validate the accuracy of the nomogram. Furthermore, the performance of the nomogram was validated via calibration curves and decision curve analysis (DCA). R software (version 4.2.1) was used to perform statistical analyses. The comparegroups, glmnet, survival, rms, dcurves, survminer, ranger, survex, and randomForestSRC packages were primarily utilized.

## Results

### Clinlabomics-score

**Fig. 2:**
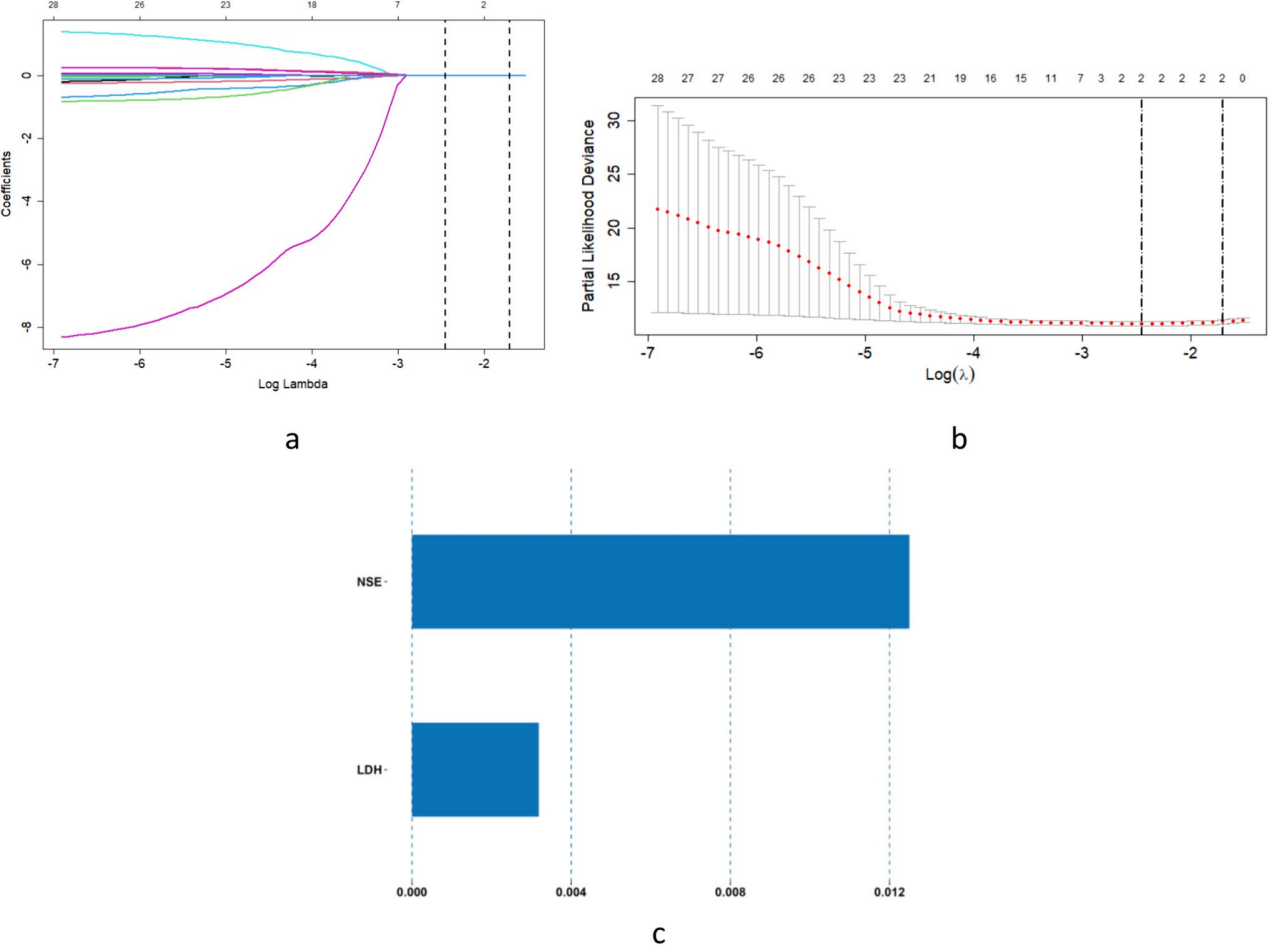
LASSO analysis of the laboratory test variables: **a** coefficient path graph; **b** cross-validation curve using tenfold cross-validation; **c** bar chart of the coefficients of the variables

In this study, we analyzed the laboratory test results of all patients and established clinlabomics-score for them. We included the first laboratory test results of the patients before treatment. Table 1 summarizes the baseline characteristics of the training and validation sets. The variables in the training set were analyzed using LASSO. Figure 2a illustrates the coefficient path diagram, and Fig. 2b illustrates the cross-validation curve. When *λ* = min, two non-zero coefficient variables were selected, namely neuron-specific enolase (NSE) and lactose dehydrogenase (LDH). Figure 2c illustrates a bar chart for the correlation coefficients. The clinlabomics-score formula was obtained using coefficient weighting as follows:

**Table 1:** Statistical characteristics of the laboratory test results for the patients in the training and validation sets

Variables		Total (*n* = 428)	Training set (*n* = 299)	Validaton set (*n* = 129)	*P* value
WBC, 10^9^	Median [Q1.Q3]	6.64 [5.58;7.89]	6.64 [5.59;7.83]	6.51 [5.54;8.12]	0.761
LYM, 10^9^	Median [Q1.Q3]	1.80 [1.47;2.20]	1.80 [1.47;2.19]	1.85 [1.50;2.23]	0.855
NEU, 10^9^	Median [Q1.Q3]	4.08 [3.25;5.05]	4.08 [3.34;4.84]	4.08 [3.14;5.40]	0.644
MONO, 10^9^	Median [Q1.Q3]	0.45 [0.34;0.57]	0.45 [0.34;0.58]	0.45 [0.35;0.57]	0.984
EOS, 10^9^	Median [Q1.Q3]	0.10 [0.06;0.19]	0.10 [0.06;0.19]	0.10 [0.06;0.18]	0.990
BASO, 10^9^	Median [Q1.Q3]	0.03 [0.02;0.04]	0.03 [0.02;0.04]	0.03 [0.02;0.04]	0.669
RBC, 10^12^	Median [Q1.Q3]	4.64 [4.38;5.02]	4.64 [4.40;5.02]	4.61 [4.34;5.02]	0.340
HGB, g/L	Mean (SD)	142.42 (14.57)	142.46 (14.17)	142.32 (15.50)	0.926
PLT, 10^9^	Median [Q1.Q3]	261.00 [217.00;310.00]	266.00 [220.50;321.50]	251.00 [215.00;300.00]	0.106
ALT, U/L	Median [Q1.Q3]	17.75 [12.79;25.00]	17.70 [12.90;24.00]	18.20 [12.40;27.60]	0.819
AST, U/L	Median [Q1.Q3]	20.00 [17.00;24.00]	20.00 [17.00;24.00]	20.00 [17.00;24.00]	0.729
GGT, U/L	Median [Q1.Q3]	26.50 [19.00;39.20]	27.00 [20.00;40.00]	24.00 [17.00;36.00]	0.100
LDH, U/L	Median [Q1.Q3]	188.00 [165.00;213.00]	189.00 [165.00;214.50]	184.00 [165.00;206.00]	0.316
ALP, U/L	Median [Q1.Q3]	95.00 [79.00;115.00]	96.00 [81.00;115.00]	93.00 [75.00;115.00]	0.259
TP, g/L	Median [Q1.Q3]	70.70 [67.50;74.30]	70.70 [67.50;74.45]	70.70 [67.50;73.80]	0.407
ALB, g/L	Median [Q1.Q3]	41.80 [39.68;43.73]	41.80 [39.55;44.00]	41.80 [39.90;43.00]	0.714
PALB, g/L	Median [Q1.Q3]	252.00 [214.00;295.00]	252.00 [214.00;292.50]	253.00 [215.00;303.00]	0.375
GLU, mmol/L	Median [Q1.Q3]	5.10 [4.70;5.60]	5.10 [4.70;5.60]	5.10 [4.70;5.50]	0.711
NA, mmol/L	Median [Q1.Q3]	140.00 [138.75;142.00]	140.00 [138.00;142.00]	140.00 [139.00;142.00]	0.563
K, mmol/L	Median [Q1.Q3]	4.10 [3.80;4.30]	4.10 [3.80;4.30]	4.00 [3.80;4.30]	0.659
CA, mmol/L	Median [Q1.Q3]	2.30 [2.20;2.40]	2.30 [2.20;2.40]	2.30 [2.20;2.40]	0.565
PT, s	Median [Q1.Q3]	11.10 [10.60;11.70]	11.10 [10.60;11.70]	11.10 [10.70;11.70]	0.509
APTT, s	Median [Q1.Q3]	27.50 [25.80;28.92]	27.50 [25.80;28.75]	27.50 [26.00;29.60]	0.456
FIB, g/L	Median [Q1.Q3]	3.30 [2.69;4.15]	3.30 [2.80;4.26]	3.30 [2.60;3.91]	0.079
TT, s	Median [Q1.Q3]	16.70 [16.00;17.20]	16.70 [16.00;17.20]	16.70 [16.10;17.30]	0.582
CEA, μg/L	Median [Q1.Q3]	3.06 [1.76;8.05]	3.06 [1.72;7.76]	3.09 [1.89;8.91]	0.698
NSE, ng/ml	Median [Q1.Q3]	16.01 [13.33;20.24]	16.01 [13.21;20.35]	16.01 [13.52;19.76]	0.971
SCC, ng/ml	Median [Q1.Q3]	0.80 [0.60;0.90]	0.80 [0.60;0.95]	0.80 [0.60;0.90]	0.687

*WBC* ^white blood cell count, *LYM*lymphocyte, *NEU*neutrophil, *MONO*monocyte, *EOS*eosinophil, *BASO*basophil, *RBC*red blood cell count, *HGB*hemoglobin, *PLT*platelet, *ALT*alanine aminotransferase, *AST*aspartate aminotransferase, GGT gamma−glutamyl transpeptidase, *LDH*lactate dehydrogenase, *ALP*alkaline phosphatase, *TP*total protein, *ALB*albumin, *PALB*prealbumin, *Glu*glucose, *Na*sodium, *K*potassium, *Ca*calcium, *PT*plasma prothrombin time, *APTT*activated partial thromboplastin time, *FIB*fibrinogen, *TT*thrombin time, *CEA*carcinoembryonic antigen, *NSE*neuron−specific enolase, *SCC*squamous cell carcinoma antigen^

Clinlabomics-score = 0.0866915 + 0.003186822 × LDH + 0.012498152 × NSE. Then, the clinlabomics-score of all patients in the training and validation sets were separately calculated. Furthermore, the clinlabomics-score of the patients with and without BM in the two datasets were determined. In both datasets, we observed statistically significant differences between patients with and without BM (Table 2).

**Table 2:** Statistical significance of the clinlabomics-score of the patients with and without BM

		Training set (*n* = 299)		*P* value	Validation set (*n* = 129)		*P* value
		BM	Non-BM		BM	Non-BM	
Clinlabomics-score	Medican [Q1,Q3]	0.99 [0.89,1.24]	0.85 [0.77,0.95]	< 0.001	0.94 [0.87,1.07]	0.83 [0.78,0.93]	< 0.001

### Patient characteristics

No statistically significant differences between the training and validation sets were observed when the clinlabomics-score and nine other clinical variables were simultaneously analyzed (P > 0.05). The median age of the 428 patients was 64 years. Furthermore, 51.64% of the patients were males, 39.25% had a smoking history, 30.84% had a pathologic type of SCLC, and 20.09% had a family history of cancer. During initial diagnosis, 33.18% exhibited lymph node metastasis and 23.36% did not exhibit lymph node metastasis. Table 3 summarizes the detailed information of both datasets.

**Table 3:** Statistical results of the clinlabomics-score and clinical variables of the patients in the training and validation sets

Variables		Total (*n* = 428)	Training set (*n* = 299)	Validation set (*n* = 129)	*P* value
Lymph node metastasis	*n* (%)				0.336
No		286 (66.82)	195 (65.22)	91 (70.54)	
Yes		142 (33.18)	104 (34.78)	38 (29.46)	
Non-lymph node metastasis	*n* (%)				0.735
No		328 (76.64)	231 (77.26)	97 (75.19)	
Yes		100 (23.36)	68 (22.74)	32 (24.81)	
Age, years	Median [Q1,Q3]	64.00 [57.00;69.00]	63.00 [57.00;69.00]	65.00 [57.00;69.00]	0.935
Sex	*n* (%)				0.982
Male		221 (51.64)	155 (51.84)	66 (51.16)	
Female		207 (48.36)	144 (48.16)	63 (48.84)	
Smoking	*n* (%)				1.000
No		260 (60.75)	182 (60.87)	78 (60.47)	
Yes		168 (39.25)	117 (39.13)	51 (39.53)	
Family history of cancer	*n* (%)				0.245
No		342 (79.91)	234 (78.26)	108 (83.72)	
Yes		86 (20.09)	65 (21.74)	21 (16.28)	
Pathologic type	*n* (%)				0.939
SCLC		132 (30.84)	91 (30.43)	41 (31.78)	
Adenocarcinoma		251 (58.64)	177 (59.20)	74 (57.36)	
LSCC		45 (10.51)	31 (10.37)	14 (10.85)	
Diameter, mm	Median [Q1,Q3]	30.50 [20.00;45.00]	31.00 [20.00;45.00]	29.00 [20.00;46.00]	0.915
Position	*n* (%)				0.764
Left upper lobe		103 (24.07)	72 (24.08)	31 (24.03)	
Left inferior lobe		85 (19.86)	62 (20.74)	23 (17.83)	
Right upper lobe		125 (29.21)	89 (29.77)	36 (27.91)	
Right middle lobe		39 (9.11)	24 (8.03)	15 (11.63)	
Right inferior lobe		76 (17.76)	52 (17.39)	24 (18.60)	
Clinlabomics-score	Median [Q1,Q3]	0.89 [0.80;1.03]	0.90 [0.80;1.03]	0.89 [0.80;1.02]	0.553

*SCLC* small cell lung cancer, *LSCC* lung squamous cell carcinoma

### Independent predictive factors

**Fig. 3:**
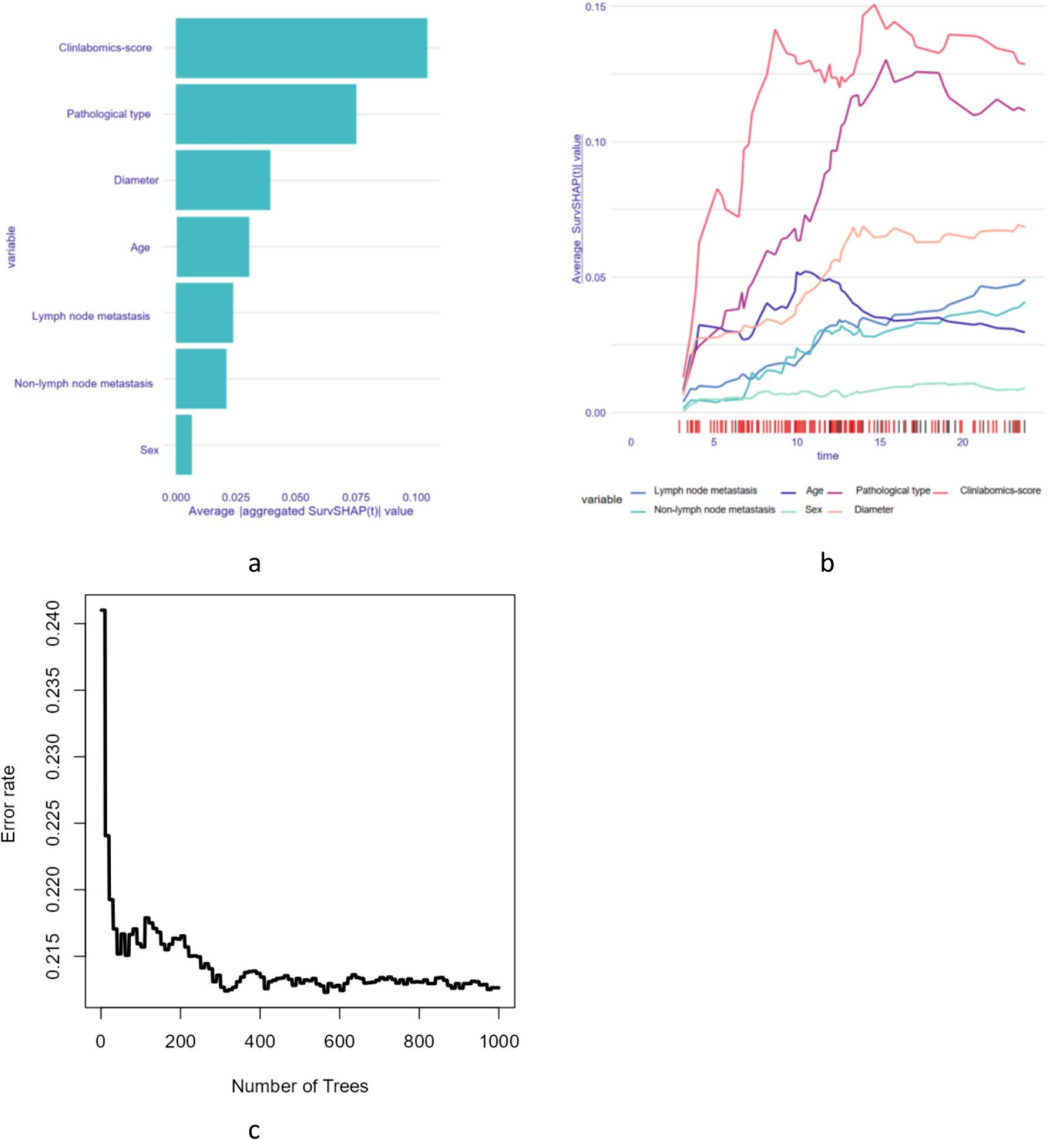
RSF analysis results: **a** bar chart showing the average aggregated SurvSHAP values of the variables; **b** time-average SurvSHAP value curve; **c** error rate of RSF

The average aggregated SurvSHAP values of the variables were determined using RSF. As a result, seven factors associated with BM were identified (Fig. 3a). Figure 3b illustrates the time-average SurvSHAP value curve. Figure 3c shows the error rate of RSF, when the number of trees is greater than 400, the error rate tends to be stabilized. These seven factors were included in multivariate Cox regression analysis (Table 4), identifying pathological type, diameter, lymph node metastasis, non-lymph node metastasis, and clinlabomics-score as the independent predictive factors for BM in primary lung cancer.

**Table 4:** Multivariate Cox regression analysis of the factors associated with BM in primary lung cancer

Factors	HR	95% CI	P value
Clinlabomics-score	2.383	1.676–3.388	< 0.001
Pathologic type			
SCLC			
Adenocarcinoma	0.325	0.209–0.506	< 0.001
LSCC	0.424	0.214–0.843	0.014
Diameter	1.012	1.002–1.021	0.016
Age	0.995	0.974–1.017	0.645
Lymph node metastasis			
No			
Yes	2.05	1.400–3.003	< 0.001
Non-lymph node metastasis			
No			
Yes	1.868	1.277–2.732	0.001
Sex			
Male			
Female	0.805	0.534–1.212	0.298

*SCLC* small cell lung cancer, *LSCC* lung squamous cell carcinoma

### Nomogram construction

**Fig. 4:**
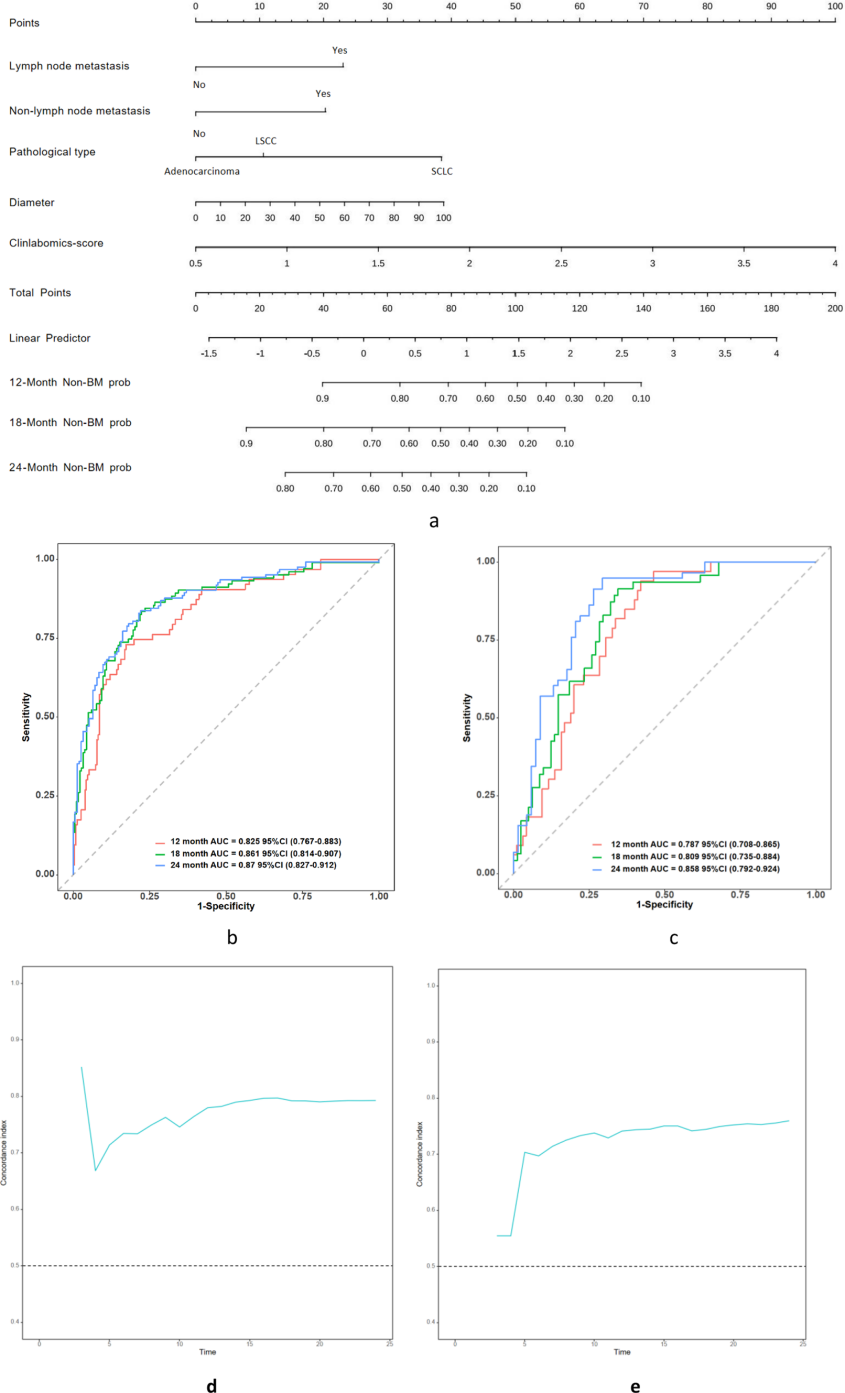
The establishment and validation of nomogram: **a** nomogram for predicting Non-BM Prob in patients with primary lung cancer at 12, 18, and 24 months after initial diagnosis; **b** ROC curve of the training set; **c** ROC curve of the validation set; **d** time-bootstrap C index of the training set; **e** time-bootstrap C index of the validation set

Next, a nomogram (Fig. 4a) was constructed using the five independent predictive factors selected via multivariate Cox regression analysis. This nomogram was used to predict Non-BM Prob in primary lung cancer at 12, 18, and 24 months after initial diagnosis. When using the nomogram, we first determined the point for each independent predictive factor separately; then, we added the points and drew a vertical line down at the total point. The intersections with the three predicted time points were Non-BM Prob.

### Performance and validation of the nomogram

The discrimination ability of the nomogram was validated using AUC. As demonstrated in Fig. 4b, in the training set, the AUC of the nomogram at the three time points (12, 18, and 24 months) were as follows: 0.825 (95% CI 0.767–0.883), 0.861 (95% CI 0.814–0.907), and 0.870 (95% CI 0.827–0.912), respectively. Furthermore, as demonstrated in Fig. 4c, in the validation set, the AUC of the nomogram at the three time points (12, 18, and 24 months) were as follows: 0.787 (95% CI 0.708–0.865), 0.809 (95% CI 0.735–0.884), and 0.858 (95% CI 0.792–0.924), respectively. Moreover, the bootstrap method was utilized to calculate the bootstrap C-index of the training and validation sets via 200 self-sampling attempts. The bootstrap C-index of the training and validation sets was 0.7931 (95% CI 0.7517–0.8284) and 0.7672 (95% CI 0.7092–0.8037), respectively. Figure 4d and Fig. 4e are the curve of the bootstrap C index of the training set and the validation set over time, respectively. It can be found that the model has a stable and accurate prediction performance between 12 and 24 months. Collectively, the findings suggest the good predictive performance of the nomogram.

**Fig. 5:**
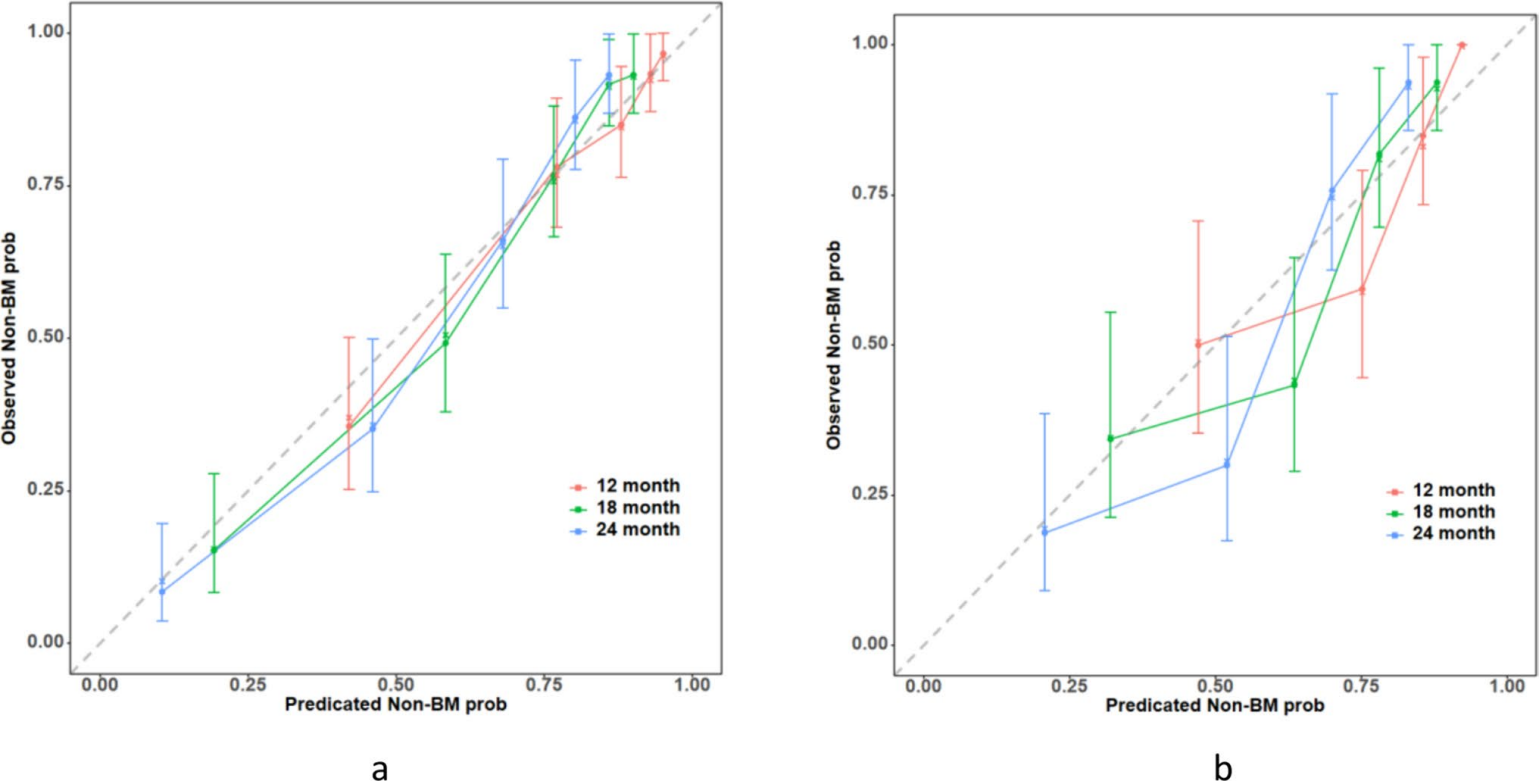
Calibration curves: **a** the curve of the training set; **b** the curve of the validation set

Figure 5a, b illustrate the calibration curves for the training and validation sets at three time points. The calibration curves revealed the high consistency of the nomogram between the predicted and observed Non-BM Prob in the training and validation sets.

**Fig. 6:**
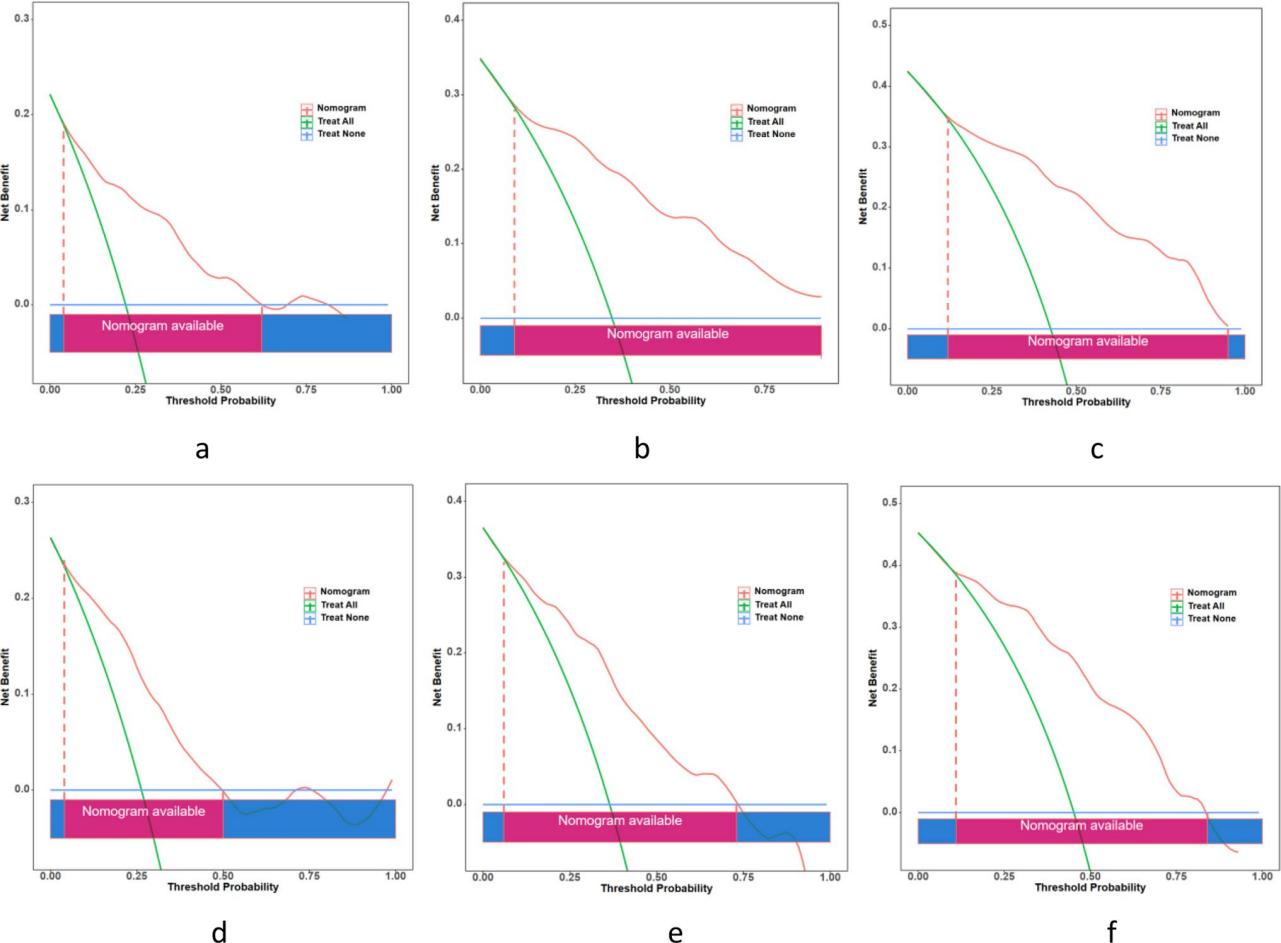
DCA results: the 12-, 18-, and 24-month curves of the training set (**a–c**) and the 12-, 18-, and 24-month curves of the validation set (**d–f**)

Furthermore, DCA was performed to explore the net benefit that the nomogram can achieve in practical clinical applications. The threshold probabilities of generating net benefits at 12, 18, and 24 months were 0.04–0.62, > 0.09, and 0.12–0.95, respectively, in the training set (Fig. 6a–c). On the other hand, in the validation set, the threshold probabilities of generating net benefits at 12, 18, and 24 months were 0.04–0.50, 0.06–0.73, and 0.11–0.84, respectively (Fig. 6d–f). These results suggest the practicality and clinical decision-making effectiveness of the nomogram.

**Fig. 7:**
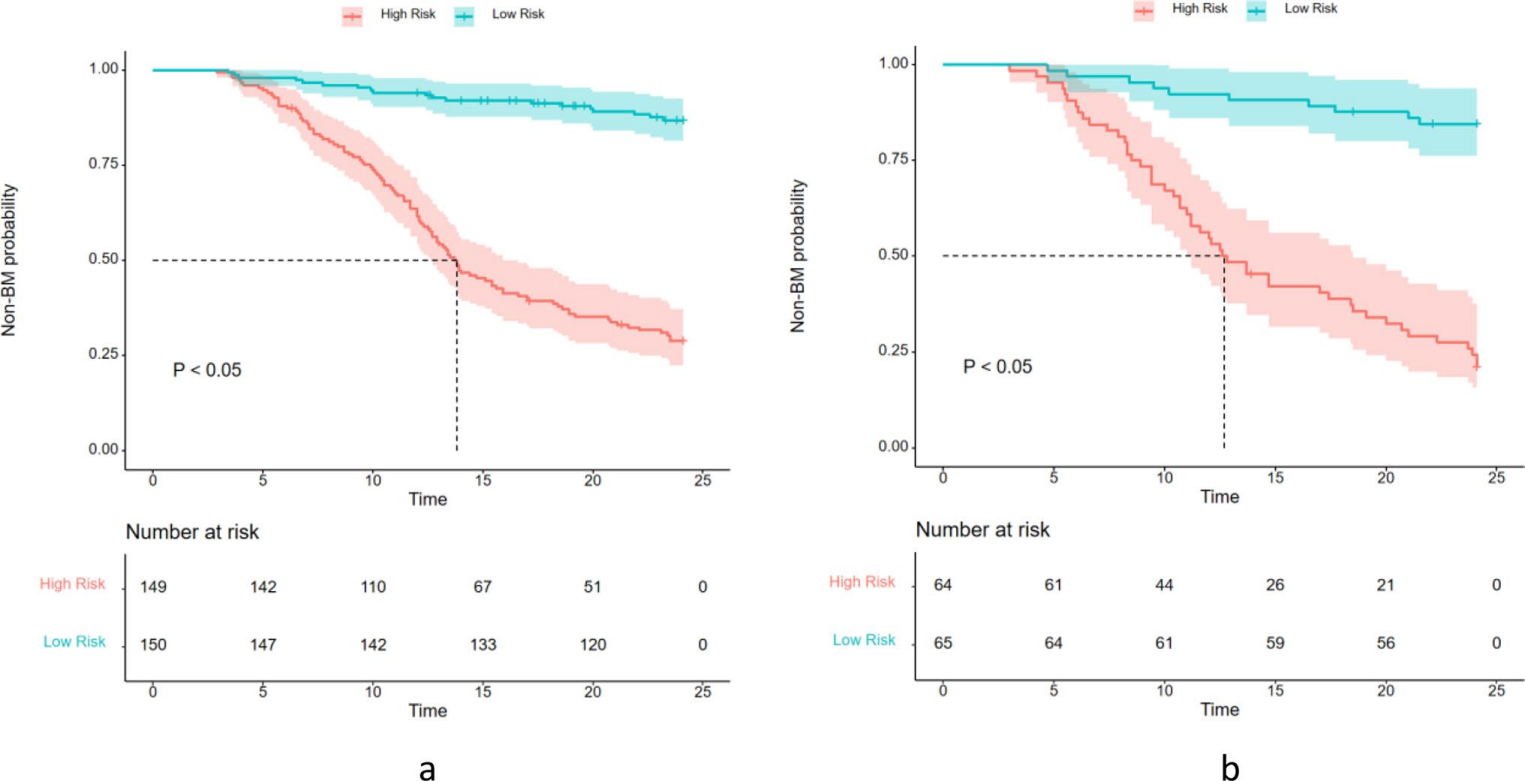
Kaplan–Meier curves of the high- and low-risk groups: **a** the Kaplan–Meier curve of the training set; **b** the Kaplan–Meier curve of the validation set

The nomoscore for each patient were calculated based on the nomogram. Then, using the median value of nomoscore, the patients of both datasets were divided into two groups: high- and low-risk groups. Then, Kaplan–Meier curves were used to validate the effectiveness of the nomogram. Figure 7a, b illustrate the Kaplan–Meier curves for the training and validation sets. The P values for both sets were < 0.05, indicating the good performance of the nomogram.

## Discussion

In this study, we retrospectively analyzed the data of 428 patients who were diagnosed with primary lung cancer. The endpoint event was BM. RSF and multivariate Cox regression analysis revealed that patients with lymph node metastasis, non-lymph node metastasis, pathological type of SCLC, large tumor diameter, and higher clinlabomics-score frequently exhibit a higher BM risk. We used these five independent predictive factors to construct a nomogram to predict Non-BM Prob in patients with primary lung cancer at three time points. In the validation set, the Bootstrap C-index was 0.7672 (95% CI 0.7092–0.8037), and the AUC at the three time points was 0.787 (95% CI 0.708–0.865), 0.809 (95% CI 0.735–0.884), and 0.858 (95% CI 0.792–0.924), respectively. This suggests the good accuracy of the nomogram. In addition, calibration curve, DCA and Kaplan–Meier curves indicated the high credibility of the nomogram.

A study based on the SEER database revealed primary site, residence type, age, histologic type, race, and extrabrain metastasis as the independent risk factors for BM; furthermore, SCLC had the highest BM incidence. Based on these findings, a nomogram with a C-index of 0.61 was constructed [15]. Using NSE, pathologic type, number of metastatic lymph nodes, and tumor grade, Zhang et al. constructed a nomogram for predicting BM in NSCLC; the C-index of their developed nomogram was 0.74 (95% CI 0.67–0.82) [16]. Using pathologic type, pN staging, and pT staging, Won et al. constructed a nomogram for predicting BM in NSCLC. The calculated bootstrap C statistics for predicting BM in 2 and 5 years were 67.0% and 67.4%, respectively [17]. Sun et al. analyzed 517 patients and constructed a nomogram using clinical and pathologic factors and treatment modes for predicting BM probability after the complete resection of IIIA-N2 NSCLC. The AUC of the nomogram was 0.767 (95% CI 0.758–0.777) [18].

In the present study, we did not analyze the laboratory test results and clinical variables together. Instead, we applied the concept of clinlabomics to determine clinlabomics-score, forming a new predictive indicator. This approach will entirely improve the role of laboratory tests in disease prediction and effectively decrease the concern of collinearity between laboratory test results and clinical data. We selected two independent predictive factors in laboratory tests, namely LDH and NSE, using machine learning methods.

As an intracellular enzyme, LDH can affect the cancer progression via the cancer glycolysis pathway or other tumor-promoting effects. Therefore, it can be utilized as a vital diagnostic marker, therapeutic target, and prognostic marker for cancers [19, 20]. Xie et al. established a K-RAS- or EGFR-driven mouse model of NSCLC and reported that LDH-A inactivation can decrease cancer occurrence and regression. In addition, LDH-A is important for the survival and proliferation of cancer cells [21]. Liu et al. have reported that serum LDH can be used to predict BM probability in SCLC after chest radiotherapy and prophylactic cranial irradiation [22]. Moreover, LDH levels can affect the treatment outcomes of patients with lung cancer. In a retrospective study involving 593 patients with NSCLC who received platinum-based treatment, researchers observed that CEA and LDH levels can be used to predict the responses of patients with advanced NSCLC to platinum-based chemotherapy [23]. Wei et al. conducted a retrospective study and reported the higher survival rate of patients with NSCLC and high CRP levels but low LDH levels after receiving anti-PD-1/PD-L1 treatment, thereby affecting patient prognosis [24]. Another study has revealed that LDH can guide patients with lung cancer before or during the treatment; furthermore, it can be used as a prognostic marker for lung cancer to predict disease progression [25].

NSE is a serum tumor marker that is primarily present in the cytoplasm of neurons and neuroendocrine cells. It can be utilized to distinguish SCLC from NSCLC, predict prognosis, and guide the treatment [26, 27]. Yuan et al. used reassigned CEA, CYFRA21-1, and NSE to establish diagnostic and prognostic models of lung cancer. In the test set, the AUC of the diagnostic and prognostic models was 0.84 and 0.8, respectively; this indicates that serum tumor markers such as NSE play important roles in lung cancer diagnosis and prognosis [28]. Li et al. have reported that NSE is one of the indicators to predict brain parenchymal metastasis in NSCLC [29]. In addition, they identified the role of NSE in predicting BM in SCLC [30].

At present, studies on the diagnostic models of BM in lung cancer BM primarily focus on predicting the BM state at the current time point, with studies on nomograms that can predict the BM state at multiple time points being scarce. Therefore, we established a nomogram that can predict BM occurrence at different time points to facilitate dynamic decision-making among clinical doctors to intervene early and improve the treatment effectiveness and quality of life of patients. In addition, through a literature search, we identified that studies have not used clinlabomics-score, providing a reference and control for subsequent clinlabomics research.

This study has some limitations and areas for improvement. First, the nomogram was constructed and validated at the same medical institution, without an external validation set; therefore, evaluating the performance of the nomogram at other institutions is challenging. Second, slight bias may exist in patient characteristics owing to the limitations of a single institution and a small sample size. Third, owing to the possible differences in the testing methods and standard settings in each laboratory, the application effect of clinlabomics in clinical settings is worth investigating. In addition, BM was observed only for 24 months. If conditions permit, a longer observation period can be used to obtain better prediction and application effects for the nomogram. Lastly, owing to missing data, we failed to obtain treatment information for most patients; therefore, we did not explore the relationship between treatment regimens and BM occurrence. In summary, we will identify methods to address the abovementioned challenges in future research to further improve the performance of the nomogram.

## Conclusion

We established a nomogram by combining clinlabomics-score and clinical data to predict BM risk in primary lung cancer at 12, 18, and 24 months after initial diagnosis. We identified lymph node metastasis, non-lymph node metastasis, pathologic type, tumor diameter, and clinlabomics-score as the independent predictive factors for BM. The nomogram is convenient and accurate for determining BM status, facilitating clinical decision-making, assessing disease progression, and providing early intervention to improve the quality of life of patients.

## Data Availability

The datasets generated during and/or analyzed during the current study are available from the corresponding author on reasonable request.
